# Control of Emission Color of High Quantum Yield CH_3_NH_3_PbBr_3_ Perovskite Quantum Dots by Precipitation Temperature

**DOI:** 10.1002/advs.201500194

**Published:** 2015-07-14

**Authors:** He Huang, Andrei S. Susha, Stephen V. Kershaw, Tak Fu Hung, Andrey L. Rogach

**Affiliations:** ^1^ Department of Physics and Materials, Science & Centre for Functional Photonics (CFP)City University of Hong KongHong KongChina; ^2^ Department of Physics and Materials ScienceCity University of Hong KongHong KongChina

**Keywords:** CH_3_NH_3_PbBr_3_, perovskite, photoluminescence, precipitation, quantum dots

## Abstract

**Emission color controlled, high quantum yield CH_3_NH_3_PbBr_3_ perovskite quantum dots** are obtained by changing the temperature of a bad solvent during synthesis. The products for temperatures between 0 and 60 °C have good spectral purity with narrow emission line widths of 28–36 nm, high absolute emission quantum yields of 74% to 93%, and short radiative lifetimes of 13–27 ns.

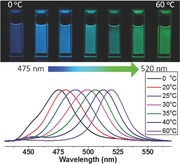

Nanometer‐sized II–VI, III–V, and IV–VI semiconductor particles with size‐ and surface‐dependent properties, often termed as colloidal quantum dots (QDs), have been extensively studied for more than 30 years meanwhile,^1^ with a variety of applications explored such as in photovoltaics, biosensors, light emitting diodes (LEDs), and so on.^2^ Perovskite semiconductors on the other hand have already shown great potential in many areas.^3^, ^4^, ^5^, ^6^ Amongst them, organic–inorganic hybrid perovskites, such as organo‐lead halide perovskites (with general structure APbX_3,_ where A represents a small molecule organic amine, and X is a halide) can be prepared at low cost and show useful optical properties, as well as superior electrical conductivity.^4^, ^7^, ^8^, ^9^ Specifically, the iodide and bromide based CH_3_NH_3_PbX_3_ have drawn extensive attention for their breakthrough performance in thin film mesoporous oxide solar cells where power conversion efficiencies of nearly 20% have already been reported.^7^, ^9^, ^10^, ^11^, ^12^, ^13^, ^14^, ^15^, ^16^ The same material also shows great potential in lasing and LEDs applications.^17^, ^18^, ^19^, ^20^

CH_3_NH_3_PbX_3_ perovskite nanoparticles were first explored as components of mesoporous titania based solar cells in 2009.^10^ But not until 2012 was high luminescence attributable to such nanoparticles observed in mesoporous material, in that case synthesized by a template method.^21^ Pérez‐Prieto and co‐workers used the capacity of medium length alkyl chain organic ammonium cations (octylammonium bromide or octadecylammonium bromide) to produce colloidal perovskite QDs of 6 nm in diameter with ≈20% emission quantum yield (QY).^22^ The same group further enhanced their QY to 82% by fine‐tuning precursors and ligands.^23^ Muthu and Nagamma recently used this method to prepare nanoparticles for explosive picric acid detection application.^24^ Chen et al. synthesized nano‐ and microsized CH_3_NH_3_PbI_3_ perovskite particles by using mild heating of CH_3_NH_3_I and PbI_2_ in different polar solvents.^25^ Zhang et al. used a ligand‐assisted re‐precipitation technique to produce brightly luminescent colloidal CH_3_NH_3_PbBr_3_ QDs with QY up to 70%.^26^ Kovalenko and co‐workers reported another QD perovskite system, CsPbX_3,_ which exhibited not only compositional bandgap engineering, but also size‐tunability of their bandgaps with reaction temperature.^27^ Zhu et al. synthesized different morphologies for CH_3_NH_3_PbX_3_ QDs by using different capping ligands and pointed out the potential of low‐dimensional perovskite semiconductors for applications in light‐emitting, imaging, and tracking devices.^28^ Notably, besides APbX_3_ organo‐lead halide perovskite nanoparticles, there are also particles in the form of A_2_PbX_4_, A_4_PbX_6_, and A(A′)_2_Pb_2_X_7_ which also show light emission.^29^, ^30^, ^31^, ^32^, ^33^

In this Communication, we demonstrate the size‐tunability of the bandgap of narrow size dispersion CH_3_NH_3_PbBr_3_ perovskite QDs (PQDs) by using temperature to exert control over the ligand‐assisted reprecipitation process. The fine control this allows, also beneficially improves their emission QY, with values ranging from 74% up to 93% with the corresponding emission peaks covering the range from 475 to 520 nm.

The reprecipitation technique employed here has been used for organic nanoparticles, polymer dots and organolead halide perovskites via the similar solvent mixing scheme.^32^, ^33^, ^34^, ^35^, ^36^, ^37^, ^38^, ^39^ In the present system, good solvent (*N*‐dimethylformamide, DMF) containing precursors and long‐chain ligands (typically with 18 carbon atom chains, which is longer than previously reported by the Pérez‐Prieto and co‐workers^22^ and Zhang et al.^26^ were injected into toluene as a bad solvent under vigorous stirring. As suggested by Zhang et al.,^26^ the crystallization process of perovskite nanoparticles is controlled by the supersaturation induced by the solubility change upon solvent mixing. Amines control the kinetics of crystallization, which mainly contributes to the size control of PQDs, while oleic acid plays an important role in suppressing the QD aggregation effects and contributes to their colloidal stability. The use of the interplay between good solvent and bad solvent follows Papavassiliou's method.^33^ One important and major factor in our modification of this approach is the temperature of the bad solvent (with greater thermal mass) where the lower volume precursor is injected into. By modifying the temperature, we successfully control the size of resulting PQDs with consequent tuning of their emission peak. In a typical synthesis, PbBr_2_, oleylamine, oleic acid, and CH_3_NH_3_Br were codissolved in DMF to form a clear transparent solution. A certain amount of this precursor solution was quickly injected to the toluene which was precooled or heated under vigorous stirring to selected set points in the temperature range of 0–60 °C. A light yellow to yellow green solution was observed immediately and signaled the formation of the PQDs. The solution was centrifuged at 14.5 krpm to remove much larger yellow particles, and a bright supernatant solution was further analyzed.

The emission peaks of as‐prepared PQDs can be tuned in the region of 475–520 nm by changing the temperature of the bad solvent during reprecipitation. **Figure** **1**a shows the gradual red shift of PQD emission (left to right) under UV lamp illumination. Optical absorption and emission spectra of selected CH_3_NH_3_PbBr_3_ PQDs (Figure 1b,c) show narrow emission linewidths of 28–36 nm, indicating the narrow size distributions. The absorption spectra of the PQDs also show a band edge red shift with increasing synthesis temperature. The absorption spectrum trend near the band edge is similar to that seen in other colloidal QD systems such as CdSe and CdTe: when the particles are small enough the absorption spectra show a peak at the band edge indicating that the oscillator strength of the lowest lying excited state is higher than that of the next highest excitation energies. For larger sizes the oscillator strength of these lower lying bands becomes much more comparable and the band edge peak is replaced by a less distinct shoulder. The PQD samples have a small 10–20 nm Stokes shift between the absorption edge and the emission peak (Figure 1c) which is consistent with a direct exciton recombination process.

**Figure 1:**
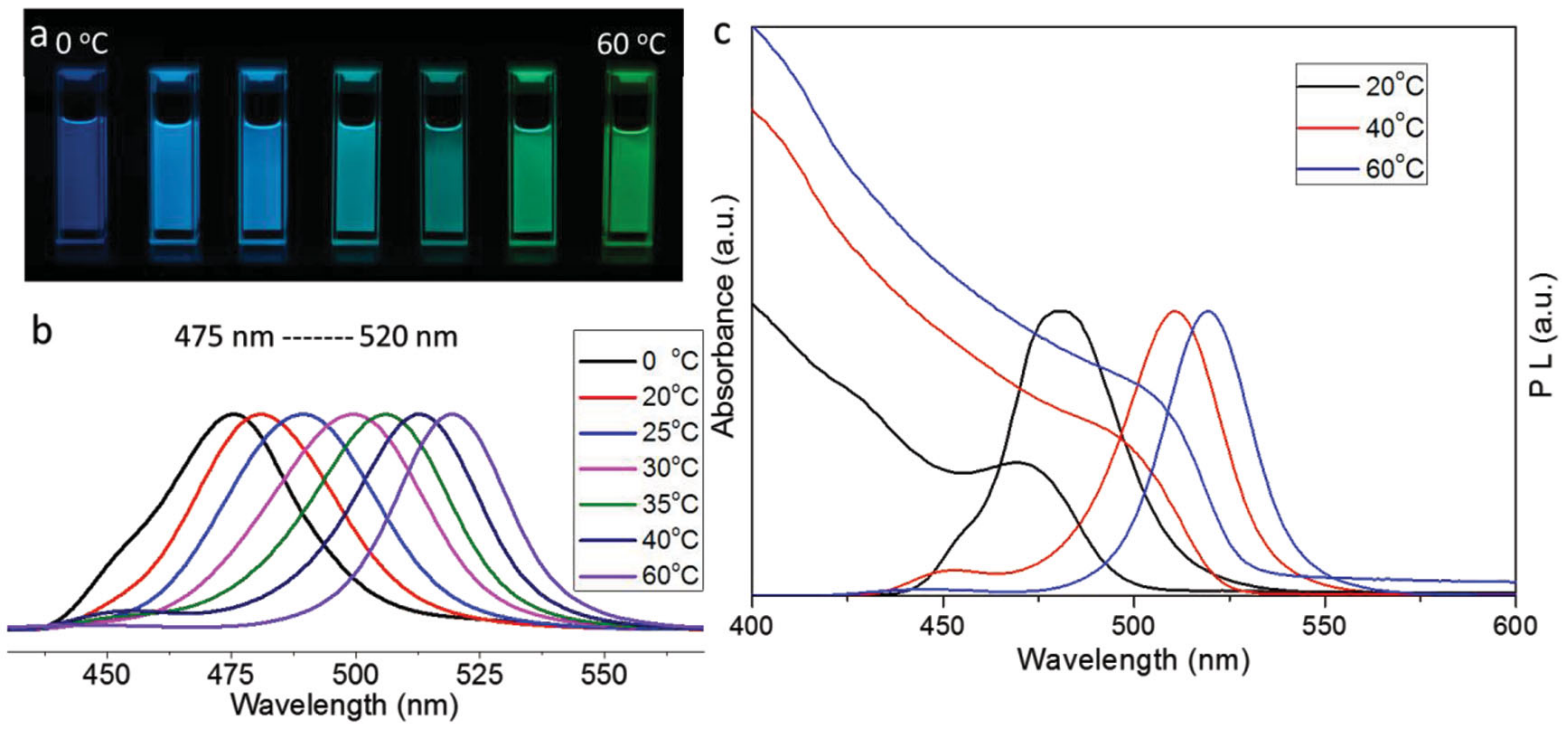
CH_3_NH_3_PbBr_3_ PQDs exhibit a size‐tunable bandgap with narrow and bright emission: a) photograph of colloidal solutions in toluene under UV lamp excitation (λ = 365 nm); b) PL spectra tunability over the range of wavelengths indicated; c) optical absorption spectra with the respective PL spectra for the three samples synthesized at different precipitation temperatures as indicated.

The XRD patterns for a freeze dried 30 °C PQD sample and the corresponding discarded large particle precipitate from the same preparation batch are shown in **Figure** **2**. Due to trace amounts of DMF, in the solutions, simple direct evaporation of solvent from solutions on the grid will lead to the formation of recrystallized bulk perovskite due to the slower drying time of DMF relative to that of toluene. Removal of the solvents by sublimation from a frozen solid prevents this recrystallization process from occurring. The lines at the bottom of the figure are the reference positions for the specified reflection measured for bulk powder samples with the same stoichiometries.^40^ Broadening of the characteristic lines is consistent with small particle size broadening according to the Debye–Scherrer expression, further confirming the presence of QD perovskite particles. To further confirm the crystal structure, we also characterized the discarded bulk precipitates (Figure 2) and found the diffraction peaks to be sharper indicating effectively bulk perovskite larger sized particles.

**Figure 2:**
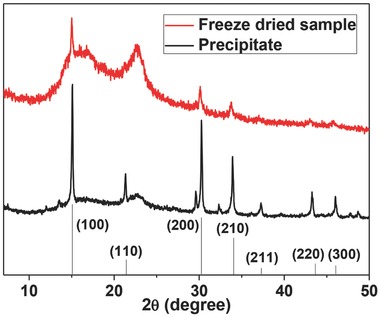
XRD patterns of CH_3_NH_3_PbBr_3_ PQDs (freeze dried sample) and the discarded precipitate from the same batch. Reference lines for specified reflections derived from literature values^40^ are shown.

**Figure** **3**a shows a typical transmission electron microscopy (TEM) image of CH_3_NH_3_PbBr_3_ PQDs. We calculated the average diameters of 0, 30, 60 °C PQDs to be 1.8, 2.8, 3.6 nm, respectively. The corresponding emission peaks are located at 2.61, 2.48, and 2.38 eV, respectively, and are consistent with a size dependent bandgap and emission. The HRTEM image for the 60 °C sample is shown in Figure 3b along with one selected particle (Figure 3c) and the corresponding fast Fourier transform (FFT) image of the same region (Figure 3d). The interplanar distances of 1.33 and 1.50 Å corresponding to the (420) and (400) crystal faces, respectively, with angle of roughly 25°, are consistent with the structure identified by XRD. The nanocrystal selected in Figure 3c shows clear lattice fringes, but the orientation is such that several of the crystal facets of the nonspherical PQD are observed simultaneously, leading to a complex FFT image with several overlapping patterns in Figure 3d.

**Figure 3:**
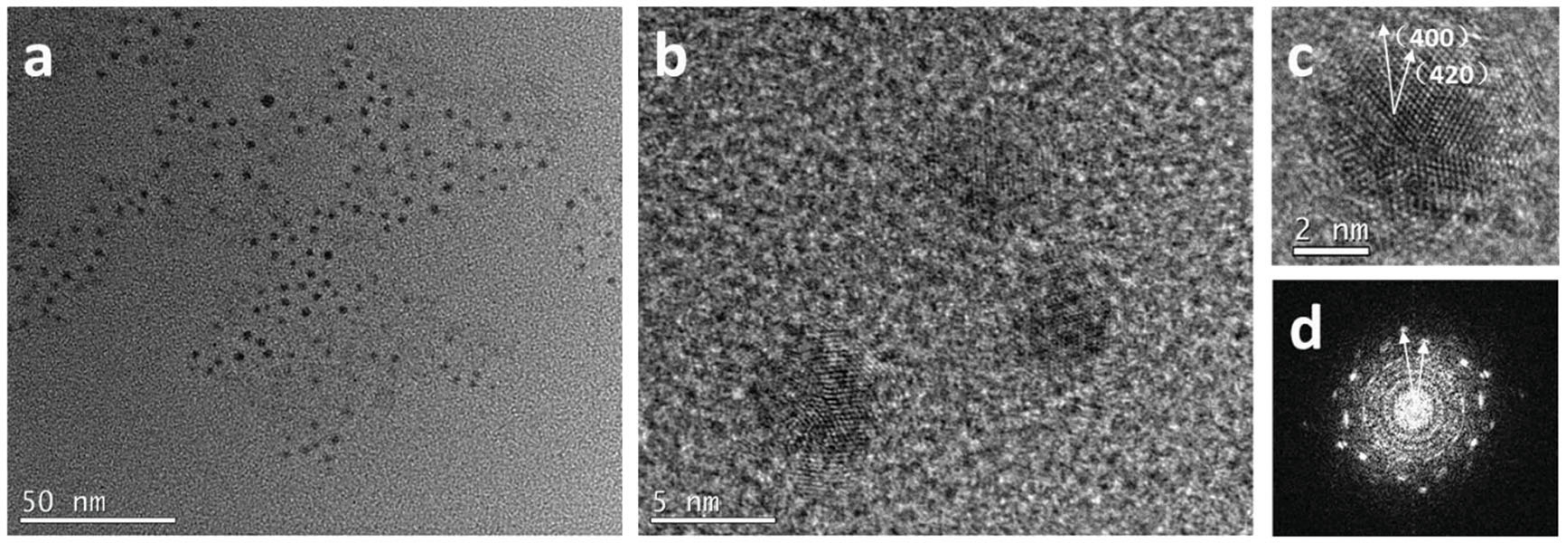
TEM image of a) representative CH_3_NH_3_PbBr_3_ QDs (60 °C); b) the corresponding HRTEM image; c) a single selected particle at high magnification; and d) the FFT pattern for the same image region.

The absolute photoluminescence (PL) QYs of the PQDs were measured using a fluorescence spectrometer equipped with an integrating sphere and excitation at a wavelength of 405 nm. The latter was chosen to match the excitation wavelength used in PL lifetime measurements. The QYs of the series of PQD materials started at 74% for the lowest synthesis temperature (0 °C) and steadily increased, reaching as high as 93% for the 60 °C sample. To best of our knowledge, the 93% QY obtained from the 60 °C synthesis is the highest value reported to date, the previous best being 82%.^23^ We have also estimated the PL QYs of the sample from the 60 °C synthesis at excitation wavelengths of 350, 380, 400 nm, and have found PL QYs very similar—in the range of 88% to 92%. To further understand the mechanism behind the outstanding QYs, time‐resolved PL decay measurements were carried out.

Representative time‐resolved PL decay spectra of CH_3_NH_3_PbBr_3_ PQDs are shown in **Figure** **4**. The PL decays were fitted to triexponential decay functions and average PL lifetimes in the range of 13–27 ns were derived. Radiative lifetimes generally decrease slightly as the emission shifts to longer wavelengths whilst nonradiative lifetimes increase as the precipitation temperature is increased. According to Fermi's Golden Rule, the radiative recombination rate, 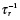, is given as(1)
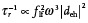
where *f*_lf_ is a local field factor accounting for the difference between the dielectric constant of the nanoparticles and the surrounding medium, *ω* is the transition frequency, and *d*_eh_ is the dipole transition matrix element due to the overlap of the electron and hole wavefunctions for the radiative recombination process. Thus, if nothing else, a slight increase in radiative lifetimes according to *ω* ^3^ might be expected in the absence of any other factors. For the change in PL peak wavelengths observed (475 to 520 nm) the radiative lifetime might increase by around 24%. The other factors, *f*_lf_ and *d*_eh_, will be sensitive to the PQD stoichiometry. Zhang et al.^26^ have shown that below 10 nm and especially below 5 nm diameters, the Br/Pb ratio for CH_3_NH_3_PbBr_3_ PQDs increases from the bulk value of slightly over 3 to 6 for the smallest (around 0.5 nm) particles studied. Increasing the Br content on reducing the nanoparticle diameter would lead to an increase in optical permittivity, thereby reducing the local field factor and the recombination rate, and increasing the radiative lifetime for small particles. Similarly, a surfeit of anions at the surface of smaller particles would favor dilation of the electron wavefunction into the surface region of the PQDs whereas the hole wavefunction would remain more localized in the nanoparticle core. Thus for smaller particles the electron–hole overlap integral and therefore *d_eh_* is likely to be lower than for larger more bulk‐like material. Therefore radiative recombination would be slower for smaller particles. These two factors evidently outweigh the *ω* ^3^ dependence and lead to a beneficial increase in the radiative rate as the particle size increases. The reduced dilation of the electron wavefunction as the particles increase in diameter also has a favorable effect on the nonradiative recombination rate, as reduced surface localization keeps the carrier on average further from surface defect sites. Thus, although the number of potential surface defect sites per dot may be increasing as the PL shifts to longer wavelengths, the time averaged probability of the carrier being near the surface decreases, leading to longer nonradiative lifetimes. The trends in both radiative and nonradiative lifetimes together lead to the increase of QY as the PQD diameter is increased.

**Figure 4:**
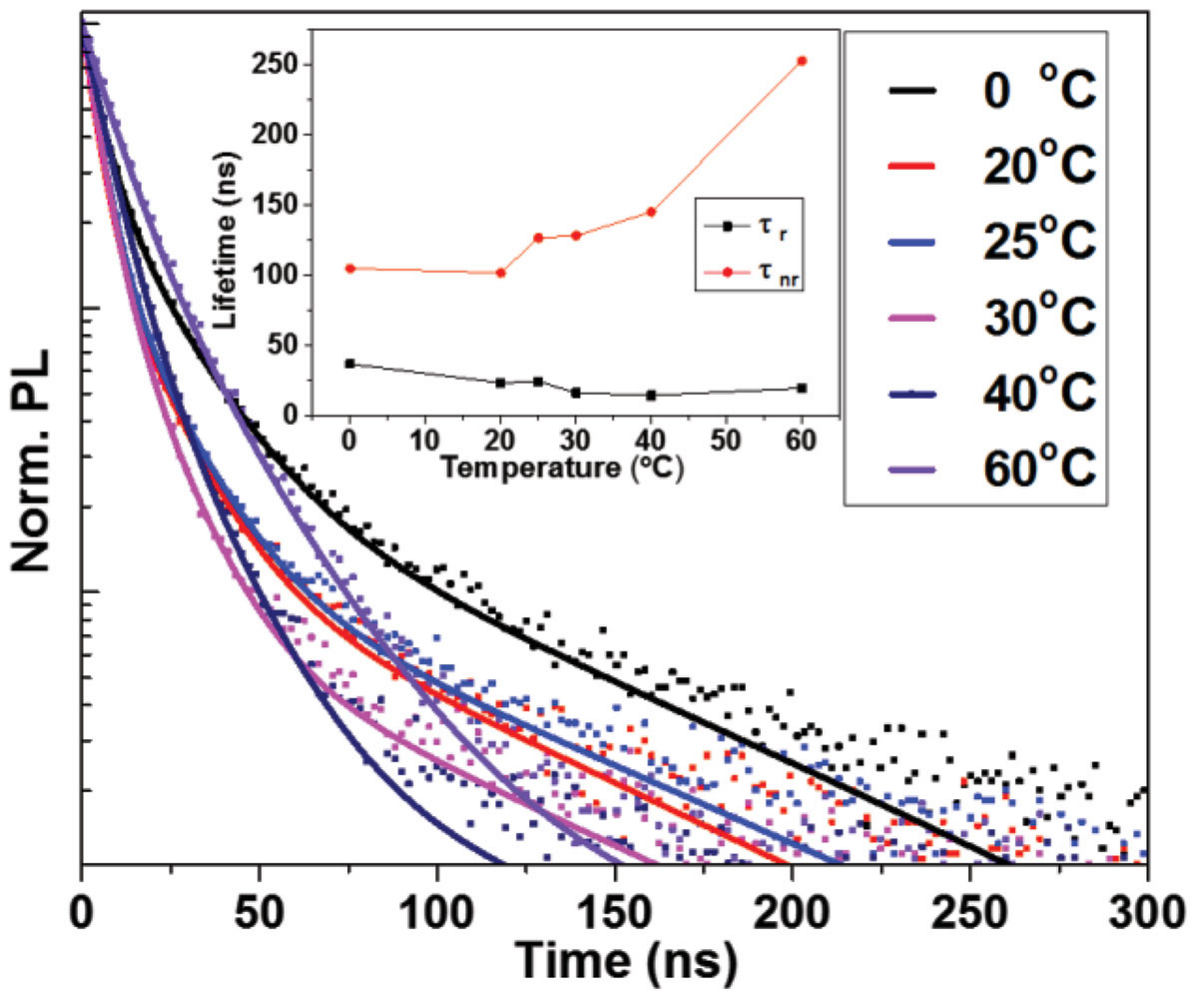
Time‐resolved PL decays and fitted curves for PQD samples grown at 0 to 60 °C, along with the trends in radiative recombination lifetime (*τ*_r_) and nonradiative recombination lifetime (*τ*_nr_) presented in inset.

For comparison, 100 ns average decay time are typically observed in CH_3_NH_3_PbBr_3_ films (PL peak at 530 nm).^41^ Due to the decreased size in our PQD materials, the average lifetime of CH_3_NH_3_PbBr_3_ QDs was greatly reduced suggesting that the PL decay of CH_3_NH_3_PbBr_3_ QDs may mainly took place through exciton radiative recombination. The relative contributions of radiative and nonradiative recombination (as shown in Figure 4 inset) to the overall decay rate were calculated from the average PL lifetimes and PL QYs (summarized in **Table** **1**). The radiative recombination lifetime (*τ*_r_) is 3–12 times faster than the corresponding nonradiative recombination lifetime (*τ*_nr_) resulting in the high PL QYs observed.

**Table 1:** Quantum yields (QY), average decay lifetimes (*τ*_avg_), radiative recombination lifetimes (*τ*_r_), and nonradiative recombination lifetimes (*τ*_nr_) of CH_3_NH_3_PbBr_3_ QDs synthesized at different temperatures

	QY [%]	*τ*_avg_ [ns]	*τ*_r_ [ns]	*τ*_nr_ [ns]
PQDs 0 °C	74	27.2	36.8	104.8
PQDs 20 °C	81	19.0	23.4	101.7
PQDs 25 °C	84	20.5	24.5	126.7
PQDs 30 °C	89	14.5	16.3	128.2
PQDs 40 °C	91	13.1	14.4	145.1
PQDs 60 °C	93	18.2	19.6	252.6

In conclusion, we have shown the first demonstration of a size‐tuned bandgap in CH_3_NH_3_PbBr_3_ PQDs by using temperature to control a facile reprecipitation technique. The emission peak range of the PQDs obtained is 475–520 nm. The PL of the PQDs is characterized by narrow emission line widths of 28–36 nm, and an outstandingly high absolute QY of 74% to 93% which we believe is the highest reported until now. The latter is a consequence of the relatively short radiative lifetimes of 13–27 ns in comparison with nonradiative lifetimes of 100 ns and longer. The improvement of PL QY may be the contribution of both, the use of surface ligands with longer chains as compared to previous work of Perez‐Prieto and co‐workers^22^ and Zhang et al.,^26^ and the higher crystallinity of the core resulting from the use of higher precipitation temperature. These very high quantum yield perovskite nanoparticles offer an outstanding potential for optoelectronic applications. Already, for example, Zhu et al have successfully demonstrated the use of related perovskite nanowires for optically excited lasing.^20^

## Experimental Section

*Materials*: All reagents were used as received without further purification: PbBr_2_ (98%, Aldrich), methylamine (33% in absolute ethanol, Sigma‐Aldrich), HBr (48%, Sigma‐Aldrich), oleylamine (80%–90%, Accuchem), oleic acid (AR, Accuchem), DMF (99%, Sigma‐Aldrich), toluene (99.7% GC, Sigma‐Aldrich)

*Synthesis of CH_3_NH_3_Br*: CH_3_NH_3_Br was synthesized with methylamine and HBr. Methylamine (33% in absolute ethanol) was precooled to 0 °C, under vigorous stirring and HBr added slowly. The as‐prepared solution was stirred for 2 h. The reacted solution was rotary evaporated and the powder was redissolved in ethanol and washed three times with diethyl ether for precipitation and dried under vacuum.

*Fabrication of CH_3_NH_3_PbBr_3_ QDs*: In a typical synthesis of CH_3_NH_3_PbBr_3_ QDs, 0.4 mmol PbBr_2_, 0.1 mL oleylamine, 1 mL oleic acid, and 0.32 mmol CH_3_NH_3_Br were codissolved sequentially with ultrasonic agitation after each step in 10 mL DMF to form a clear transparent solution. 5 mL aliquots of toluene were precooled or heated to set temperatures ranging from 0 to 60 °C and 0.5 mL of precursor solution was quickly injected into each with vigorous stirring. Strong PL emission was observed immediately after injection. After centrifugation at 14.5 krpm for 5 min to sediment insoluble precipitates, a bright supernatant solution was obtained. The solution was diluted 10 times for further analysis. The synthetic yield of CH_3_NH_3_PbBr_3_ PQDs is limited due to the formation of bulk material by‐products along with the desired small‐sized QDs and further decreased with the increase of synthesis temperature.

*Characterization*: Powder X‐ray diffraction (XRD) patterns were taken on a Philips X‐Pert X‐ray diffractometer using Cu Kα radiation (λ = 1.5418 Å). Transmission electron microscopy was performed on Philips CM‐20 and JEOL JEM 2100F (HRTEM). Absorption spectra were recorded using a Cary 50 UV–vis spectrophotometer (Varian). PL spectra were measured on a Cary Eclipse (Varian) model and also a FLS920P fluorescence spectrometer (Edinburgh Instrument) equipped with a photon counting photomultiplier (R928P, Hamamatsu), with a 450 W xenon arc lamp as the excitation source for steady state and integrated quantum yield measurements. The PL quantum yield, defined as the ratio between photons emitted and absorbed by the sample, was determined by an absolute method using an integrating sphere with its inner face coated with BENFLEC (Edinburgh Instrument), fitted to the spectrofluorimeter. The average PL decay lifetimes were measured using a 405 nm, 49 ps pulse width laser and a time correlated single photon counting system. Decay curves were fitted to multiple‐exponential decay curves and the average lifetimes were calculated as 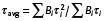, where *B_i_* are the amplitudes of the component decay times *τ_i_*.
